# Perineal hygiene in recurrent urinary tract infections - protective or predisposing?

**DOI:** 10.1186/2047-2994-4-S1-P263

**Published:** 2015-06-16

**Authors:** DPCKA Lal

**Affiliations:** 1 Surgery, National Hospital of Sri Lanka, Colombo, Sri Lanka

## Introduction

Recurrent urinary tract infection is a common problem in pediatric clinical practice. Unsatisfactory perineal hygiene considered to be a main predisposing factor in theoretical background as the etiology of the condition is opportunistic microrganisms found in the perineal commensal microbiota. The impact of perineal hygiene on the occurence of urosepsis is not well studied in Sri Lankan community.

## Objectives

The objective of this study is to evaluate the role of different perineal hygiene practices in recurrent urinary tract infections among Sri Lankan paediatric population.

## Methods

45 female patients with recurrent urinary tract infections and age matched 45 controls were evaluated for perineal hygiene practices. All subjects were in the age of 1 to 5 years. Recurrent urinary tract infection was defined as 3 or more episodes of treated urosepsis within last one year period. Chi square test was used with the significance level of 0.05 for statistical analysis.

## Results

Recurrent urinary tract infections were ecountered in 13, 04 and 28 children in the categories of washing with soap and water, washing with water and no washing respectively. Recurrent urinary tract infections were not found in 02, 21 and 22 children in the categories of washing with soap and water, washing with water and no washing respectively.

## Conclusion

There is a statistically significant difference (P < 0.05) of UTI occurrence among different perineal hygiene practices. Washing with water seems to be protective whereas washing with soap and water seems to be predisposing.

Washing with water probably remove pathogenic microorganisms thereby preventing infections and washing with soap probably remove considerable amount of commensal organisms there by promoting colonization by pathogens. A prospective randomized controlled study with a bigger sample is recommended for more reliable information.

## Disclosure of interest

None declared.
